# Sensory Blurring in Nociplastic Pain: The Role of Descending Inhibitory Dysfunction and Gut–Brain Axis Alterations in Older Adults

**DOI:** 10.3390/geriatrics11030071

**Published:** 2026-06-16

**Authors:** Takahiko Nagamine

**Affiliations:** 1 Sunlight Brain Research Center, Hofu 7470066, Yamaguchi, Japan; tnagamine@outlook.com; 2 Shoshido Geriatric Health Services Facility, Hofu 7470825, Yamaguchi, Japan

**Keywords:** descending pain inhibitory system, sensory refinement, nociplastic pain, aging, lateral inhibition, gut–brain axis, central sensitization, sex differences

## Abstract

**Background**: Inhibitory processes in the nervous system are traditionally conceptualized as suppressive mechanisms; however, their fundamental role is the refinement and optimization of sensory information. In nociception, this function is mediated by the descending pain inhibitory system (DPIS), which modulates nociceptive transmission at multiple hierarchical levels. Biological sex and gender-related factors significantly influence these inhibitory pathways, yet they are often overlooked in clinical frameworks. **Methods**: A narrative review was conducted using PubMed, MEDLINE, and Scopus databases, focusing on studies published between 2010 and 2025. Search terms included “descending pain inhibitory system,” “nociplastic pain,” “aging,” “sex differences,” “gender,” and “gut–brain axis.” Approximately 30 key references were synthesized. **Results**: The DPIS enhances the precision of nociceptive signals through mechanisms analogous to lateral inhibition. In chronic and nociplastic pain, this refinement process is impaired, leading to “sensory blurring.” Aging exacerbates these changes through neurochemical depletion and neuroinflammation. Crucially, this decline follows sex-specific trajectories; estrogen depletion in post-menopausal females accelerates the loss of monoaminergic inhibitory reserves, while gender-related sociocultural stressors can further disrupt top-down executive control. Additionally, alterations in the gut–brain axis signaling—modulated by sex-specific gut microbiota profiles—further disrupt inhibitory control. **Conclusions**: Chronic pain may be conceptualized as a disorder of sensory refinement rather than excessive nociceptive input. Inclusion of sex and gender as biological variables is essential for precision pain management. Therapeutic strategies should focus on restoring inhibitory precision through both central and systemic approaches, tailored to the patient’s hormonal and physiological profile.

## 1. Introduction

Pain is a complex perceptual experience that arises from the integration of peripheral nociceptive input and central processing mechanisms rather than representing a direct measure of tissue damage [1,2,3]. This distinction is formally recognized by the International Association for the Study of Pain, which defines nociplastic pain as pain arising from altered nociception without clear evidence of actual or threatened tissue damage or disease of the somatosensory system [4]. This definition underscores the importance of central modulation in shaping pain perception and highlights the need to better understand the systems that regulate nociceptive signaling [5].

Across sensory systems, inhibition plays a critical role in enhancing signal fidelity. One of the most fundamental mechanisms underlying this process is lateral inhibition, which improves contrast and sharpens spatial resolution by suppressing neighboring neuronal activity [6,7]. This principle is well established in visual and somatosensory systems, where it enables precise localization and discrimination of stimuli. Extending this concept to nociception provides a useful framework for understanding the function of the descending pain inhibitory system (DPIS) [8]. Rather than simply reducing pain signals, the DPIS can be understood as a system that refines nociceptive information by enhancing relevant signals and suppressing noise [9,10,11,12]. Within this framework, the present review introduces the concept of “sensory blurring,” defined as a functional loss of spatial and temporal summation control in nociceptive processing. In this context, sensory blurring is defined as a failure of top-down inhibitory precision, resulting in the spatial and temporal spread of nociceptive signals. Psychophysically, this is characterized by a reduction in the two-point discrimination threshold and an impairment in Conditioned Pain Modulation (CPM), where the ‘pain-inhibits-pain’ phenomenon is lost. Sensory blurring manifests clinically as diffuse, poorly localized pain that lacks clear correspondence with peripheral pathology and is a hallmark of chronic and nociplastic pain conditions [13,14]. This perspective shifts the focus from pain intensity to signal precision and provides a unifying explanation for a wide range of clinical observations.

The aim of this review is to synthesize current knowledge of the DPIS as a sensory refinement network and to explore how its dysfunction contributes to chronic pain, particularly in the context of aging. In addition, the review examines emerging evidence linking the gut–brain axis to descending pain modulation, highlighting the role of systemic factors in shaping central inhibitory processes. To ensure scientific rigor, it is critical to define the operational scope and boundaries of this narrative review. While we rely firmly on empirical clinical and preclinical data from 2010 to 2025, our goal is not merely to compile a catalog of pain symptoms. Rather, this review is scoped to synthesize these distinct datasets into a cohesive, systems-level framework—the ‘sensory blurring’ model. We explicitly acknowledge the boundary between established neurochemical evidence and the speculative hypotheses presented herein, which are intended to stimulate, rather than replace, direct empirical testing.

## 2. Materials and Methods

This narrative review was conducted to integrate current evidence on the DPIS, its role in sensory processing, and its clinical implications. To ensure methodological transparency and minimize selection bias, a structured literature search was conducted across PubMed, MEDLINE, and Scopus for articles published between 2010 and 2025. Search terms included “descending pain inhibitory system,” “nociplastic pain,” and “aging,” with additional mechanistic terms such as “lateral inhibition” and “NMDA receptor.” Inclusion criteria prioritized peer-reviewed meta-analyses, systematic reviews, and key experimental studies that discussed descending modulation in geriatric or age-related contexts. Articles were excluded if they focused primarily on acute post-operative pain or pediatric populations. The reference list was expanded to 55 sources to ensure a comprehensive overview of the shifting paradigms in pain science. While this remains a narrative synthesis, this structured approach provides a more rigorous foundation for the proposed “sensory blurring” framework.

The review prioritized systematic reviews, meta-analyses, and key experimental studies, while also incorporating conceptual and hypothesis-driven articles to support theoretical integration. Both human and animal studies were included to provide complementary perspectives. Reference lists of selected articles were manually screened to identify additional relevant publications. Approximately 30 references were included in the final synthesis.

## 3. The Neurobiology of Sensory Refinement in Pain

The DPIS consists of a hierarchical network involving the periaqueductal gray (PAG), the RVM, and descending projections to the dorsal horn of the spinal cord [15]. These structures are embedded within a broader network that includes cortical and limbic regions, allowing cognitive and emotional factors to influence pain modulation. Traditionally, the DPIS has been viewed as a system that suppresses nociceptive transmission; however, this perspective does not fully capture its functional complexity [16].

The DPIS operates as a sensory refinement network, functioning to optimize the signal-to-noise ratio of nociceptive input. While anatomically distinct from the localized microcircuits found in the retina, the DPIS provides a functional analogy to lateral inhibition at a systemic level. It refines sensory “borders” by suppressing non-salient background noise. Much of the granular evidence for this refinement—specifically the “On-cell” and “Off-cell” firing patterns in the RVM—is derived from rodent electrophysiology. In these animal models, inhibition is not just “turning off” pain; it is sharpening the contrast between noxious stimuli and harmless background activity. However, in humans, this system is more adaptive and state-dependent than classical lateral inhibition, heavily modulated by the prefrontal cortex and emotional state [17,18,19]. Without such refinement, nociceptive input would be diffuse and difficult to interpret, leading to maladaptive responses. Lateral inhibition and the DPIS share commonalities in information refinement, but differ in reaction time and other aspects, as shown in Table 1. Lateral inhibition is largely “automatic” and hard-wired to improve the resolution of what we see and feel [20]. The descending pain system is highly adaptive, influenced by emotions, stress (stress-induced analgesia), and cognitive factors. While vision and touch rely heavily on fast-acting amino acid transmitters (GABA/Glycine), the pain inhibitory system utilizes a “cocktail” of monoamines (Serotonin, Norepinephrine) and neuropeptides (opioids) to provide longer-lasting modulation [21].

**Figure 1:**
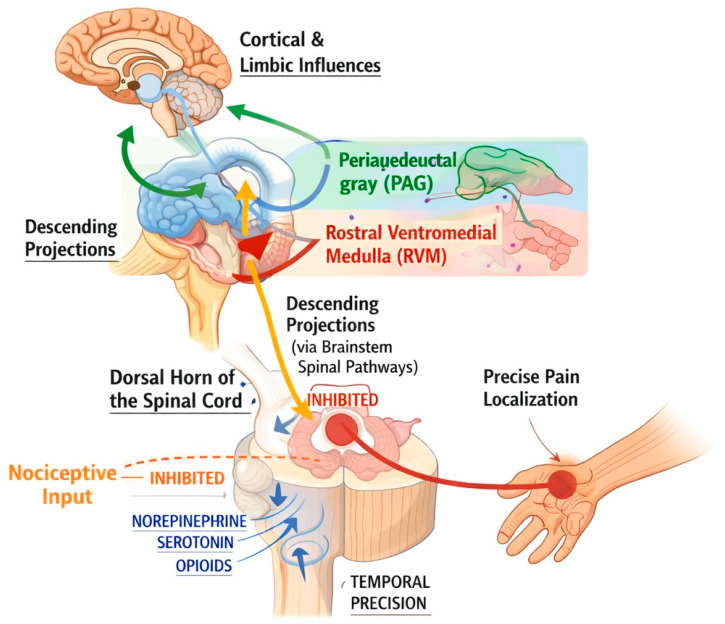
Conceptual model of the descending pain inhibitory system as a sensory refinement network. The periaqueductal gray (PAG) and rostral ventromedial medulla (RVM) modulate spinal nociceptive transmission through mechanisms analogous to lateral inhibition, enhancing spatial and temporal precision.

In the DPIS, at the level of the rostral ventromedial medulla (RVM), this refinement process is mediated by distinct neuronal populations known as on-cells and off-cells. On-cells facilitate nociceptive transmission, whereas off-cells inhibit it [22]. The dynamic balance between these two populations functions as a tuning mechanism that regulates the gain of the pain signal [23,24]. In a well-functioning system, this balance allows for rapid adaptation to changing conditions, ensuring that pain remains a precise and contextually appropriate signal (Figure 1).

## 4. Dysfunction in Chronic and Nociplastic Pain

In chronic pain conditions, the refinement function of the DPIS becomes impaired, resulting in a fundamental alteration of nociceptive processing. Rather than enhancing signal fidelity, the system becomes dysregulated, leading to amplification and persistence of pain [25]. This dysfunction occurs at multiple levels, including network connectivity, neurochemical signaling, and cellular mechanisms [26]. At the network level, reduced connectivity between the prefrontal cortex and the PAG diminishes top-down control [27], limiting the brain’s ability to regulate incoming nociceptive signals. At the neurochemical level, depletion of monoamines such as serotonin and norepinephrine reduces inhibitory capacity. At the cellular level, activation of NMDA receptors contributes to central sensitization, increasing neuronal excitability and promoting the persistence of pain [28]. These changes result in sensory blurring, characterized by diffuse and poorly localized pain. This phenomenon reflects a breakdown of inhibitory contrast mechanisms analogous to the loss of edge detection in visual systems when lateral inhibition is impaired. Importantly, nociplastic pain can be understood within this framework as a disorder of sensory refinement rather than excessive nociceptive input. Impairment of conditioned pain modulation—the psychophysical measure of the DPIS—further supports this interpretation, as it demonstrates a reduced ability of the system to regulate incoming signals through “pain-inhibits-pain” mechanisms [29].

**Figure 2:**
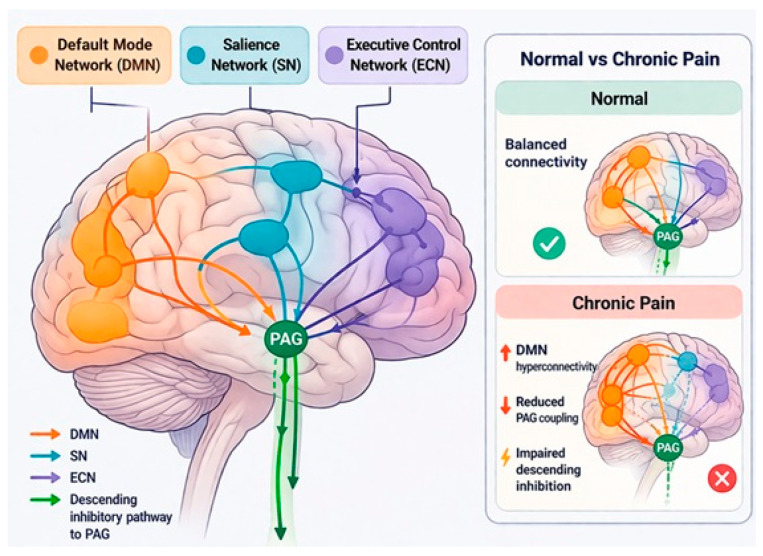
Changes in the connectivity of large-scale brain circuits and descending pain inhibitory systems in chronic pain. Disruptions include dlPFC-amygdala hyperconnectivity and S1-NAcc coupling, which impair the signal-to-noise optimization typically performed by the DPIS.

Beyond brainstem circuits, chronic and nociplastic pain are associated with alterations in large-scale brain networks that interact with the DPIS (Figure 2). These include the default mode network (DMN), salience network (SN), and central executive network (CEN), all of which play roles in attention, emotional processing, and cognitive control [30]. Recent research by [31] highlights that in older adults, chronic pain induces changes in resting-state functional connectivity (rsFC) that significantly surpass those caused by normal aging alone [31]. This suggests that chronic pain acts as an accelerator of network-level “blurring,” specifically affecting how executive and affective regions communicate with the DPIS [31].

A critical finding in this population is the hyperconnectivity between the right dorsolateral prefrontal cortex (dlPFC) and the left amygdala [31]. This abnormal link between executive control centers and emotional processing hubs is significantly correlated with lower pain-inhibition capacity during conditioned pain modulation tasks. Within the framework of sensory refinement, this hyperconnectivity suggests that emotional/affective noise “leaks” into the executive pathways, distracting the top-down inhibitory mechanisms of the dlPFC and preventing it from effectively engaging the PAG-RVM circuit [32]. Consequently, the system fails to suppress background nociceptive noise, further contributing to the diffuse nature of nociplastic pain. Furthermore, older adults with chronic pain exhibit increased connectivity between the primary somatosensory cortex (S1) and the nucleus accumbens (NAcc) compared to pain-free older adults [33]. This shift indicates that sensory information is increasingly “filtered” through motivational and reward-processing areas rather than being refined by purely inhibitory circuits. When the NAcc and S1 become hyper-coupled, the salience of pain may be amplified while the spatial precision of the signal is lost.

Finally, while normal aging involves a general reduction in connectivity between brain structures involved in pain inhibition, the presence of chronic pain exacerbates this disconnection. The breakdown of communication between sensory, affective, and executive structures leads to a state where the brain can no longer distinguish between salient nociceptive signals and background physiological noise. This suggests that nociplastic pain is not solely a dysfunction of brainstem circuits but a disorder of distributed neural networks where the DPIS is effectively “de-coupled” from the cognitive resources required to sharpen sensory input [34].

Beyond biological sex, gender-related factors—such as differing societal expectations for pain coping and healthcare-seeking behaviors—interact with large-scale brain networks. For instance, the hyperconnectivity between the dlPFC and amygdala observed in chronic pain may be influenced by gender-specific patterns of catastrophizing or emotional regulation strategies, which further distract top-down inhibitory control. Importantly, the integration of gender-related factors must not be viewed through a reductionist lens that equates complex sociocultural behaviors solely to static neural connectivity. Alterations in circuits like the dlPFC-amygdala coupling represent the dynamic biological embodiment of prolonged, gender-specific psychosocial stressors. For example, societal expectations regarding pain coping or hyper-vigilance chronically shape psychological traits, such as pain catastrophizing. These cognitive profiles impose a continuous load on the central executive network, thereby top-down distracting the dlPFC from its sensory refinement role and driving hyperconnectivity with affective hubs. Thus, gender in this review is conceptualized as a non-linear, multi-dimensional variable interacting with, rather than being reduced to, neural networks.

## 5. Aging and the Decline of Inhibitory Precision

Aging is associated with a progressive decline in the structure and function of the DPIS [35]. This decline is not uniform but results from the interaction of multiple processes, including neurodegeneration, neurochemical depletion, and systemic inflammation. Structural changes include reduced integrity of white matter tracts connecting cortical and brainstem regions, which impairs communication within the DPIS [35]. Neurochemical changes involve reductions in key neurotransmitters, including serotonin, norepinephrine, dopamine, and endogenous opioids, all of which are essential for effective inhibitory control. Neuroinflammation plays a particularly important role in this process. Age-related activation of microglia and increased production of pro-inflammatory cytokines create an environment that promotes neuronal hyperexcitability and central sensitization. This phenomenon, often referred to as inflammaging, further disrupts inhibitory processes and contributes to the persistence of pain [36].

The functional consequences of these changes are reflected in altered pain perception in older adults [37]. While sensitivity to mild stimuli may decrease, vulnerability to persistent and severe pain increases. Recovery from injury is often delayed, and the risk of developing chronic pain conditions is elevated. These findings suggest that aging is characterized by a loss of sensory precision rather than a simple change in sensitivity [38,39]. The decline of the DPIS in older adults is significantly modulated by biological sex, particularly through hormonal transitions. In females, the transition to menopause represents a critical window of vulnerability. Estrogen is a potent modulator of the monoaminergic systems; it enhances the synthesis and reduces the degradation of serotonin and norepinephrine. Consequently, the post-menopausal drop in estrogen levels leads to a rapid depletion of the inhibitory reserve, accelerating the transition from acute to nociplastic pain. Conversely, in males, age-related declines in testosterone are more gradual, which may correlate with a more sustained, albeit slowly diminishing, inhibitory capacity. This suggests that “sensory blurring” may have a more abrupt onset and higher prevalence in older women, requiring sex-specific timing for therapeutic interventions. Table 2 summarizes the age- and sex-related changes in DPIS and their functional effects.

## 6. The Gut–Brain Axis and Systemic Modulation of Pain

The gut–brain axis (GBA) represents a complex, bidirectional communication network linking the enteric nervous system (ENS), the vagus nerve, and central nervous system structures responsible for pain modulation. This axis serves as a critical mediator in physiological homeostasis and has emerged as a fundamental driver in the transition from acute to chronic pain states. Recent syntheses, such as those by Ho et al. (2025), highlight that the interaction between the gut and the “Pain Matrix”—a distributed network including the thalamus, insula, and anterior cingulate cortex—is pivotal in maintaining central sensitization [40]. The ENS communicates with the brainstem primarily via vagal afferents, which project to the nucleus tractus solitarius. These projections influence the activity of the PAG and RVM, the core components of the DPIS. Through these pathways, signals originating in the gut can modulate the balance between On-cells and Off-cells, thereby influencing the gain of nociceptive transmission. Dysfunction in the vagus nerve, often characterized by reduced vagal tone, impairs this inhibitory signaling, allowing peripheral signals to be amplified centrally [40].

Beyond direct neural pathways, the GBA influences the DPIS through the hypothalamic–pituitary–adrenal (HPA) axis. Chronic gut-derived stress, such as that caused by microbial dysbiosis, triggers HPA axis dysregulation, leading to sustained cortisol release and subsequent neuroinflammation [41]. This systemic endocrine shift effectively “reprograms” the DPIS by promoting a facilitatory state. Inflammatory signals, including lipopolysaccharides (LPS) crossing a “leaky gut” barrier, activate spinal and brainstem microglia. These primed microglia release pro-inflammatory cytokines that enhance NMDA receptor activity, further blurring the distinction between noxious and non-noxious stimuli [42].

**Figure 3:**
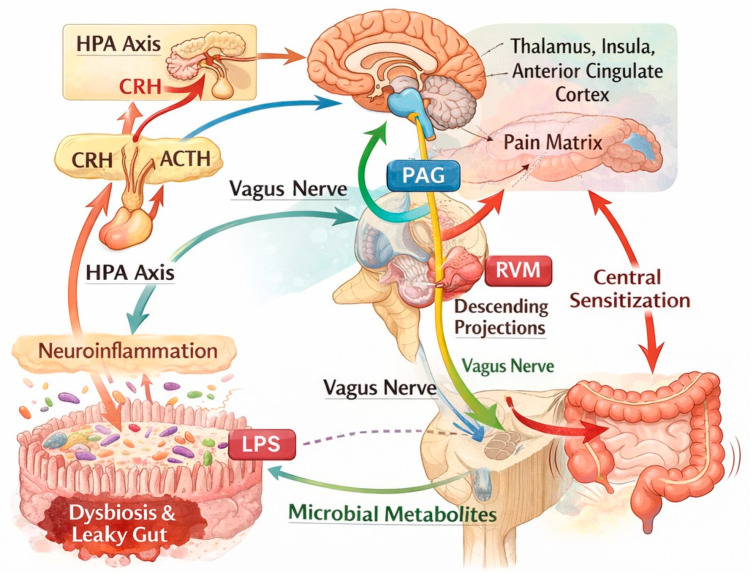
Gut–brain axis modulation of the descending pain inhibitory system. Bidirectional signaling involving the vagus nerve, HPA axis, and microbial metabolites (SCFAs) regulates the balance of the PAG-RVM circuit. Dysbiosis and leaky gut promote neuroinflammation and central sensitization, leading to a breakdown of inhibitory control.

The gut microbiota also exerts a significant influence on central processes through the production of neuroactive metabolites. Short-chain fatty acids (SCFAs), particularly butyrate, play a protective role by maintaining microglial homeostasis and strengthening the blood–brain barrier. Conversely, a loss of microbial diversity leads to reduced SCFA production, removing a key metabolic brake on neuroinflammation. Furthermore, alterations in tryptophan metabolism within the gut can divert precursors away from serotonin synthesis and toward the kynurenine pathway [43]. This reduction in available serotonin directly depletes the monoaminergic reserves of the DPIS, weakening the descending “Off-cell” drive and exacerbating nociplastic pain. These emerging mechanisms suggest that chronic pain is not merely a localized neurological failure but a systemic condition involving autonomic imbalance and gastrointestinal health. Modulating the GBA—through vagal nerve stimulation, restoration of microbial diversity, or SCFA supplementation—offers a promising therapeutic avenue to restore the inhibitory precision of the DPIS (Figure 3).

The DPIS does not operate in isolation from systemic health; it is highly dependent on the metabolic ‘tonus’ provided by the gut–brain axis. In older people, the breakdown of this axis leads to a reduction in the signal-to-noise ratio of visceral feedback. Specifically, when the DPIS is weakened, the brain’s ability to distinguish between salient nociceptive signals and background physiological ‘noise’ from the gut is compromised. This failure of sharpening leads to a state where systemic inflammation directly feeds into central sensitization, effectively ‘blurring’ the sensory boundaries of the internal milieu [44].

## 7. The Visceral-Autonomic Interface: DPIS and the Defecation Reflex

While the DPIS is primarily studied in the context of nociception, emerging evidence suggests its “sensory refinement” role extends to the modulation of autonomic reflexes. A critical example of this is the defecation reflex, which relies on a precise balance of central monoaminergic output. While AAP-induced constipation is traditionally and primarily attributed to peripheral anticholinergic and antiserotonergic effects on the gut, we propose that a central “refinement” failure may also contribute to these symptoms in older adults. Preclinical data suggest that the same monoaminergic pathways (Serotonin and Dopamine) used by the DPIS to refine pain signals are also utilized to prime the sacral parasympathetic nuclei for the defecation reflex. We hypothesize that “sensory blurring” may extend to autonomic regulation: if the DPIS fails to provide a precise excitatory drive to the bowel’s parasympathetic centers, the result is a failure of reflex initiation. This central-autonomic hypothesis does not replace established peripheral models but offers a more integrated view of why geriatric patients on AAPs experience such profound multisystem dysregulation. Recent preclinical studies suggest that the descending pathways involving dopamine (DA) and serotonin (5-HT) are vital for the initiation of the defecation reflex via the sacral parasympathetic nuclei (SPN) [45,46,47,48]. In older adults, the DPIS is often compromised by both age-related neurodegeneration and pharmacological interference. Atypical antipsychotics (AAPs), frequently prescribed to this demographic, exacerbate “sensory blurring” not only in pain pathways but in the autonomic signals required for bowel motility [47,48]. AAPs act as multi-neurotransmitter antagonists, disrupting the DA and 5-HT systems that the DPIS utilizes to prime the SPN [45,46]. When the DPIS fails to provide sufficient excitatory drive to these parasympathetic centers, the defecation reflex weakens. This suggests that the high prevalence of constipation (30–60%) in patients on AAPs is not merely a peripheral anticholinergic side effect but a central failure of the descending modulatory system. The lack of “signal clarity” in the autonomic nervous system mirrors the sensory blurring seen in nociplastic pain, where the brain cannot effectively process or respond to visceral distension signals. This dysfunction is particularly evident in the geriatric population, where the descending inhibitory system’s role in ‘sharpening’ sensory input is vital for the defecation reflex. This reflex requires a precise ‘on-demand’ activation of the sacral parasympathetic nuclei. In a healthy system, the DPIS suppresses irrelevant autonomic noise to allow this reflex signal to dominate. However, in the elderly, the reduction in monoaminergic drive—the same drive that suppresses pain—leads to a ‘blunted’ reflex. The system can no longer sharpen the signal of rectal distension, contributing significantly to the high prevalence of functional constipation in older adults.

Further complicating this refinement process is the role of GABAergic signaling. The DPIS utilizes GABA to maintain the “Off-cell” balance in the RVM. However, enhanced GABAergic inhibition, potentially influenced by sex-specific hormonal profiles, can lead to an over-suppression of the defecation reflex. This creates a physiological paradox: while the system is intended to filter noise, an overactive inhibitory state (facilitated by AAPs or aging) leads to the suppression of necessary motor outputs, contributing to the observed sex differences in gastrointestinal dysfunction among older people [49].

**Figure 4:**
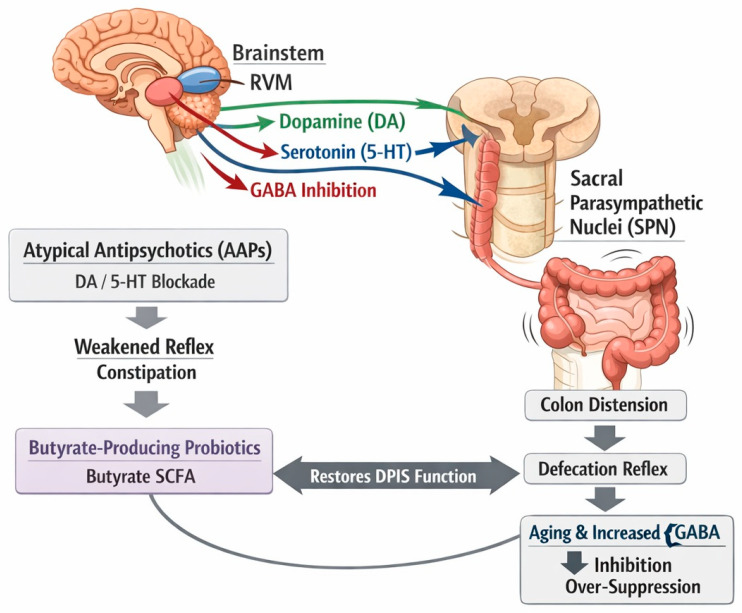
The Descending Pain Inhibitory System as a Circuit for the Defecation Reflex. The descending pain inhibitory system (DPIS) modulates the defecation reflex via monoaminergic pathways (dopamine and serotonin) that project to the sacral parasympathetic nucleus (SPN). When the DPIS functions properly, it promotes bowel motility, but dysfunction due to aging or the use of atypical antipsychotics can lead to signaling disorders and constipation in older adults. Butyrate-producing probiotics may help restore central dopaminergic tones via the gut–brain axis and improve reflex function.

A novel frontier in restoring DPIS function lies in the use of butyrate-producing probiotics. Butyrate, a SCFA, acts as more than a local energy source for colonocytes; it serves as a systemic signaling molecule that can cross the blood–brain barrier or influence the brain via the vagus nerve [50]. Evidence suggests that butyrate can restore central dopaminergic function, effectively “sharpening” the signal transmission within the DPIS [51,52]. By improving the central monoaminergic tone, butyrate-producing interventions may strengthen the compromised defecation reflex [49,50]. This highlights a bidirectional loop: a healthy microbiome supports the neurochemical integrity of the DPIS, which in turn ensures the precise execution of the autonomic reflexes necessary for gut health (Figure 4). However, to maintain strict boundary management between established evidence and hypothesis generation, this central-autonomic interface must be characterized as a highly speculative, complementary model. The primary driver of antipsychotic-induced constipation remains peripheral anticholinergic and antiserotonergic blockade. To ensure this expanded DPIS framework remains scientifically falsifiable, future clinical trials must investigate whether top-down sensory refinement directly correlates with autonomic output. Specifically, if this hypothesis holds true, geriatric patients with severe nociplastic pain and impaired Conditioned Pain Modulation (CPM) should exhibit a concomitant delay in sacral parasympathetic reflex initiation during rectal distension, independent of peripheral drug concentrations. Delineating these clinical boundaries is essential to prevent the conceptual inflation of the DPIS model.

## 8. Sex and Gender Considerations in Sensory Refinement

A comprehensive conceptual analysis of nociplastic pain must account for the distinct roles of biological sex and sociocultural gender.

### 8.1. Biological Sex

Research consistently demonstrates that females often exhibit lower conditioned pain modulation efficiency compared to males. This is likely due to sex-specific differences in mu-opioid receptor distribution within the PAG and RVM. Furthermore, microglial “priming”—a key driver of inflammaging—shows higher reactivity in females, which may exacerbate the breakdown of lateral-like inhibition and lead to more diffuse “blurring” of nociceptive signals. In the aging population, biological sex primarily modulates the DPIS through hormonal pathways. Evidence indicates that estrogen facilitates monoaminergic synthesis; thus, post-menopausal depletion in females can lead to an abrupt decline in the inhibitory “Off-cell” reserve, essentially depleting the neurochemical “budget” required for signal sharpening.

### 8.2. Gender Roles

Sociocultural factors also influence the top-down modulation of the DPIS. Gender-specific patterns in pain catastrophizing and emotional regulation can lead to the hyperconnectivity between the dlPFC and amygdala discussed in Section 4. If gendered social expectations increase emotional “noise” (affective distress), the executive centers are further distracted from their primary role in sharpening sensory input. This distraction effectively “blurs” the prefrontal-PAG filter, preventing the system from sharpening the nociceptive signal. By distinguishing between biological sex and sociocultural gender, we avoid mechanistic conflation and recognize that nociplastic pain is exacerbated by both a biological depletion of inhibitory reserves and a psychosocial distraction of top-down control. Furthermore, our framework integrates the critical roles of sex and gender by linking hormonal transitions to the systemic neurochemical ‘budget’ required for the DPIS to function. In post-menopausal females, for example, estrogen depletion represents a biological loss of monoaminergic support, yet this is compounded by gender-related psychosocial stressors that further tax the prefrontal cortex’s executive resources. By viewing the DPIS as a system with a limited neurochemical capacity, we move beyond simple neural connectivity to a more integrated model of how biological vulnerability and psychosocial load converge to exacerbate sensory blurring.

## 9. Clinical Translation: Sharpening the Blurred Signal

For the geriatrician, the concept of “sensory blurring” shifts the clinical focus from treating “excessive pain” to treating “reduced signal resolution.” When an older patient presents with diffuse, non-localized chronic pain, it should be viewed as a failure of the DPIS “filter” rather than ongoing tissue damage.

### 9.1. Pharmacotherapeutic Implications

“Signal sharpeners” like SNRIs (e.g., Duloxetine) should be prioritized over “signal suppressors” like high-dose opioids. While opioids may numb the pain, they do not restore the precision of the inhibitory system and may, in fact, contribute to further “blurring” through opioid-induced hyperalgesia [53].

### 9.2. Diagnostic Innovation

Clinicians should consider bedside measures of Conditioned Pain Modulation (CPM) to assess the “health” of the DPIS [54]. A weak CPM response indicates a “blurred” system that may benefit more from neurofeedback or exercise—both of which have been shown to “re-tune” the descending circuits—than from standard analgesics.

### 9.3. Systemic Care

Because the gut–brain axis acts as a metabolic “tuner” for the DPIS, managing gut microbiome health in older adults may be a viable strategy for stabilizing the central inhibitory environment [55]. Techniques such as conditioned pain modulation testing can serve as a functional biomarker for DPIS integrity. Future therapies might involve real-time feedback where patients utilize neurostimulation to “re-train” the top-down inhibitory pathways, effectively sharpening the focus of the PAG-RVM circuit [56].

## 10. Limitations

Despite the integrative framework proposed in this review, several limitations must be acknowledged. First, as a narrative synthesis, this work is inherently subject to selection bias; while we prioritized high-impact studies from 2010–2025, a systematic meta-analysis was not performed to quantify the effect sizes of DPIS decline in aging. Second, a significant portion of the mechanistic data regarding lateral-like inhibition in the PAG-RVM circuit is derived from preclinical animal models. While these models provide high-resolution data on On- and Off-cell activity, the degree to which these findings translate to the more complex, cognitively driven pain experiences of humans remains an area of active debate.

Furthermore, the concept of “Sensory Blurring” currently serves as a theoretical construct. While it aligns with clinical observations of diffuse nociplastic pain, it lacks a validated objective biomarker or a standardized psychophysical metric beyond the existing (and sometimes variable) conditioned pain modulation protocols. Finally, the role of the gut–brain axis in modulating the DPIS is a rapidly evolving field; many cited interventions, such as butyrate supplementation to sharpen central sensory signals, require large-scale, longitudinal clinical trials in geriatric cohorts before they can be recommended as standard clinical practice.

## 11. Conclusions

The conceptualization of the DPIS as a sensory refinement network provides a unifying framework for understanding pain across a range of conditions. By emphasizing the role of inhibition in enhancing signal fidelity, this perspective shifts the focus from suppression to optimization. The concept of sensory blurring captures the functional consequences of impaired refinement and offers a clinically relevant explanation for the diffuse and persistent nature of chronic pain. Aging further amplifies these processes by reducing inhibitory reserve and increasing susceptibility to central sensitization. The integration of the gut–brain axis into this framework highlights the importance of systemic factors in shaping central pain modulation and suggests that effective treatment may require a holistic approach that addresses both neural and peripheral components.

The DPIS plays a central role in optimizing nociceptive processing by enhancing sensory precision. Dysfunction of this system leads to sensory blurring and contributes to chronic pain, particularly in aging populations. Future research should focus on developing objective biomarkers of inhibitory function and exploring interventions that restore sensory refinement at both neural and systemic levels.

## Figures and Tables

**Table 1:** Lateral Inhibition vs. Descending Pain Inhibitory System.

Feature	Lateral Inhibition (Vision/Touch)	Descending Pain Inhibitory System
Primary Purpose	Enhancing boundaries and clarifying spatial location (sensory sharpening).	Signal-to-noise optimization and selective filtering (analgesia).
Directionality	Horizontal/Lateral: Between neighboring cells within the same circuit level.	Vertical/Descending: Top-down modulation from higher centers to the spinal cord.
Temporal Scale	Millisecond-fast (primarily ionotropic).	Prolonged and adaptive (metabotropic and neuromodulatory).
Information Focus	Spatial edges, contours, and pinpointing touch.	Salience prioritization (survival signals vs. background pain).
Key Structures	Retina (Horizontal cells), Thalamus, Somatosensory Cortex.	PAG, RVM, Spinal Dorsal Horn, Prefrontal Cortex.
Inhibitory Mediators	Primarily GABA and Glycine.	Monoamines (5-HT, NE), Endogenous Opioids, GABA.

**Table 2:** Age-related changes in the DPIS and their functional consequences.

Domain	Age-Related Changes	Functional Consequence	Sex/Gender Modulation
Structural	White matter degeneration; cortical atrophy	Reduced top-down connectivity	Earlier onset of connectivity breakdown in regions linked to estrogen signaling.
Neurochemical	Decreased 5-HT, NE, and endogenous opioids	Reduced inhibitory reserve	Estrogen depletion in females accelerates monoamine loss; testosterone decline in males is more gradual.
Cellular	Microglial priming and activation	Chronic neuroinflammation (Inflammaging)	Females often show more robust microglial reactivity to systemic gut-derived LPS.
Functional	Impaired conditioned pain modulation	Sensory blurring and poorly localized pain	Lower CPM efficiency in post-menopausal women compared to age-matched men.

## Data Availability

Data sharing is not applicable to this article as no new data were created or analyzed in this study.

## Abbreviations

The following abbreviations are used in this manuscript:

AAPs	atypical antipsychotics
CEN	central executive network
DA	dopamine
dlPFC	right dorsolateral prefrontal cortex
DMN	default mode network
DPIS	descending pain inhibitory system
ENS	enteric nervous system
GABA	gamma amino butyric acid
GBA	gut–brain axis
HPA	hypothalamic–pituitary–adrenal
5-HT	serotonin
NAcc	nucleus accumbent
NE	norepinephrine
NMDA	N-methyl-D-aspartate
PAG	periaqueductal gray
rsFC	resting-state functional connectivity
RVM	rostral ventromedial medulla
SCFA	short-chain fatty acid
SN	salience network
SPN	sacral parasympathetic nuclei
